# Elucidating the roles of the mammary and gut microbiomes in breast cancer development

**DOI:** 10.3389/fonc.2023.1198259

**Published:** 2023-08-17

**Authors:** Courtney Hoskinson, Rachel Yutong Jiang, Leah T. Stiemsma

**Affiliations:** ^1^ Department of Microbiology and Immunology, University of British Columbia, Vancouver, BC, Canada; ^2^ Natural Science Division, Pepperdine University, Malibu, CA, United States

**Keywords:** mammary microbiome, gut microbiome, breast cancer, breast tumor, breast microbiome

## Abstract

The mammary microbiome is a newly characterized bacterial niche that might offer biological insight into the development of breast cancer. Together with in-depth analysis of the gut microbiome in breast cancer, current evidence using next-generation sequencing and metabolic profiling suggests compositional and functional shifts in microbial consortia are associated with breast cancer. In this review, we discuss the fundamental studies that have progressed this important area of research, focusing on the roles of both the mammary tissue microbiome and the gut microbiome. From the literature, we identified the following major conclusions, (I) There are unique breast and gut microbial signatures (both compositional and functional) that are associated with breast cancer, (II) breast and gut microbiome compositional and breast functional dysbiosis represent potential early events of breast tumor development, (III) specific breast and gut microbes confer host immune responses that can combat breast tumor development and progression, and (IV) chemotherapies alter the microbiome and thus maintenance of a eubiotic microbiome may be key in breast cancer treatment. As the field expectantly advances, it is necessary for the role of the microbiome to continue to be elucidated using multi-omic approaches and translational animal models in order to improve predictive, preventive, and therapeutic strategies for breast cancer.

## Introduction

Breast cancer, apart from nonmelanoma skin cancer, is the most common cancer in women and affects approximately 12% of all women during their lifetimes (1, 2). Characterization of the mammary and gut microbiomes in breast cancer is well underway but much regarding the interaction of these microbiomes and their hosts begs to be clarified, with the breast tissue ecological niche only recently described (3, 4). Most recent research on the breast and gut microbiome suggests dysbiosis, an imbalance in the microflora, which may preclude the development of breast tumors (4–7). However, the mechanisms linking the microbiome to breast tumor development are still under investigation and the directionality of this relationship has yet to be resolved. Does the microbiome instigate breast cancer or is the changing tumor microenvironment responsible for alterations in microbial composition? How does the microbiome interact with its host to progress or prevent tumor development? In this review, we discuss the foundational and fundamental studies that have progressed these important areas of research, focusing on the roles of both the mammary tissue and gut microbiomes in breast cancer initiation, progression, and response to therapeutics.

## The role of the breast tissue microbiome in breast cancer

### Breast tissue compositional dysbiosis in cancerous breast tissue varies by tumor type and cancer stage

Researchers recently characterized the microbiome within human breast tissue, identifying significant differences in bacterial composition in healthy tissue relative to tumor tissue (**Table S1**) (3, 8–10). Notably, the identification of microbes within the classical tumor environment is a replicated phenomenon (11, 12), with specific intracellular bacteria being implicated in a variety of unique tumor-type environments, including *Fusobacterium nucleatum*, a microbe associated with epigenetic changes in the cancer environment, in pancreatic and breast tumors, the pathogenic and clinically-relevant *Enterobacter cloacae* in pancreatic, breast and glioblastoma multiforme tumors, and *Citrobacter freundii*, known to cause urinary tract infections, diarrhea, pneumonia, and meningitis and intracranial abscesses, in pancreatic, lung, and breast tumors (13, 14). Even inter-tumor microbiome signatures can differ in microbiome composition. One group used whole genome and transcriptome amplification and pan-pathogen microarrays to compare the microbiome within tumor subtypes, i.e., groups of tumors that are different according to histological characteristics of their respective tissues, cancer cell features, or genetic alterations (15). The researchers used hierarchical clustering to group tissues based on their microbiome signatures derived from these multi-omics techniques, with common differential organisms including increased *Actinomyces*, *Bartonella*, *Brevundimonas*, *Coxiella*, *Mobiluncus*, *Mycobacterium*, *Rickettsia*, and *Sphingomonas* within tumor tissues as compared to non-cancerous control tissue (15).

More recent studies are also beginning to elucidate breast cancer-associated bacterial dysbiosis in relation to breast cancer subtypes and breast cancer stages between populations of different races/ethnicities. This is reflective of the difference in incidence and mortality within these populations as well, as White women are more likely than Black or Asian women to develop breast cancer, but Black women experience a higher rate of mortality and worse severity of disease (16). A study comparing non-Hispanic Black and White women emphasized the complexity of the tumor microbiota (17). 16S ribosomal RNA (rRNA) gene amplicon sequencing was conducted on normal tissue (n = 8), normal adjacent tissue (normal pairs, n = 11), and breast tumors (n = 64), with a total of 13 stage 1, 24 stage II, and 19 stage III and IV breast cancer tissues analyzed by 4 subtypes of luminal A, luminal B, human epidermal growth factor receptor 2 (HER2), and triple negative breast cancer (TNBC), from non-Hispanic Black and non-Hispanic White women (17). Results from this study showed that Proteobacteria was the most abundant phylum present within all analyzed tissues, while Firmicutes, Bacteroidetes, and Actinobacteria, were less abundant (17). Regarding specific stages, the family *Ruminococcaceae* and genus *Hyphomicrobium* were abundant in stage 1 breast tumors, while stage 2 breast tumors contained increased genus *Sporosarcina* and stage 3 and 4 breast tumors showed abundance of only genus *Bosea* (17). Tumor tissues from Black women also showed a higher abundance of the genus *Ralstonia* as compared to those from White women (17). One study of Mediterranean women identified *Ralstonia* as a key breast-cancer-associated bacterium (18). Species within *Ralstonia* have markedly been implicated in various diseases, including nosocomial bloodstream infection and bacteraemia (19, 20), highlighting this bacterial group as a potential breast cancer pathogen.

A key factor supporting the colonization of bacteria in the tumor microenvironment is that this microenvironment may offer essential nutrition and oxygen for bacterial survival (21). In this way, the tumor microenvironment can provide a niche for microbial populations, but these microbial populations may also negatively respond to the enhanced metabolic activity of host tumor cells, leading to bacterial dysbiosis in breast tissue following a breast cancer diagnosis (7). Moreover, the relationship between breast tissue bacteria and cancer cells may be bidirectional; dysbiosis in breast tissue appears to support tumor progression, and tumor progression also appears to negatively affect breast tissue bacteria, suggesting the need for further research to clarify the directionality of this relationship.

### Breast tissue host-microbiome interactions in breast cancer

Characterization of the functional role of the local tumor microbiota in breast cancer is well underway. In the next sections we discuss host-breast tissue microbiome interactions in breast cancer. Furthering the concept of a bidirectional relationship between the host and mammary microbiome, we identified studies that highlight the microbiome’s influence on host cell proliferation, activity, and death, host DNA damage, and host immune function. We also identified studies that emphasize the metabolic response of the microbiome to breast tumor development.

### Breast tissue microbes and host cell proliferation, activity, and death

Researchers have observed that tumor tissue expresses lower basal levels of antibacterial response gene expression as compared to healthy breast tissue (22). However, there are a number of studies in which breast tissue microbes associate and potentially instigate changes in host cell genetic programming and cell-cycle progression, either attenuating or promoting tumor development.

Hassan et al. investigated the potential of live, heat-killed cells and the cytoplasmic fractions of *Enterococcus faecalis* and *Staphylococcus hominis* as anti-breast cancer agents (23). They used human breast cancer cell lines with estrogen, progesterone and glucocorticoid receptors, and non-malignant epithelial cell lines and treated the cells with varying amounts of each of the live, heat-killed microbes, and the cytoplasmic fractions of the bacteria (23). They evaluated cytotoxicity using the MTT assay, a colorimetric assay for assessing cell metabolic activity, morphological features of the treated cells by fluorescence microscopy, and the stage of cell cycle arrest and apoptosis by flow cytometry (23). Notably, the authors report that all three forms of the bacteria caused a significant decrease in cancer cell proliferation in a concentration- and time-dependent manner, with morphological features of apoptosis (cell death, cell shrinkage and membrane blebbing) observed, and little to no effect on normal cells (23). This suggests that these bacteria can be used as an alternative nutraceutical for breast cancer because of their non-cytotoxic effects on normal cells.

Esfandiary et al. explored the role of *Lactobacilli* in breast cancer, specifically in relation to that of the human Hypoxia-Inducible Factor (HIF)-1 (24). HIF is a major player in the body’s response to low oxygen concentrations and regulates the expression of genes implicated in homeostasis, vascularization, anaerobic metabolism, and immunological responses; its increase is also associated with increased proliferation and more aggressive breast tumor development (24). The authors analyzed the expression of *HIF-1α*, *SLC2A1*, *VHL*, *HSP90*, *XBP1*, and *SHARP1* genes from the HIF pathway in triple-negative breast cancer line cells before and after treatment with *Lactobacillus crispatus* and *Lactobacillus rhamnosus* culture supernatants by quantitative reverse transcription polymerase chain reaction (qRT-PCR) (24). The *Lactobacillus* spp. were cytotoxic to the cancer cell line and down-regulated the expression levels of the measured genes from the HIF pathway (24). This analysis indicates an important interaction and communication between commensal microbiota and host pathways involved in the homeostatic regulation of human cells.

Conversely, other groups report that specific bacteria instigate tumor growth. In a mouse study, researchers report increasing Gal-GalNAc levels as human breast cancer progresses, and that occurrence of *Fusobacterium nucleatum* genomic DNA in breast cancer samples correlates with high Gal-GalNAc levels in breast cancer cells (25). Moreover, they demonstrate that inoculation with *F. nucleatum* in the breast suppresses the accumulation of tumor-infiltrating T cells and promotes tumor growth and metastatic progression, the latter two of which could be counteracted by antibiotic treatment in mice (25). In a large cohort study, researchers integrated 16S rRNA sequencing with host tumor expression profiles in 668 breast tumor tissues and 72 non-cancerous adjacent tissues (26). They observed an increase of Proteobacteria in tumor tissues, an increase of Actinobacteria in non-cancerous adjacent tissues, and, most interestingly, an association between gene set enrichment of *Listeria* spp. and expression profiles of host genes involved in with epithelial to mesenchymal transitions (26). The study also observed that *H. influenza* correlated with genes in the G2M checkpoint, E2F transcription, and mitotic spindle assembly pathways, while *L. fleischmannii* associated with genes involved in epithelial to mesenchymal transition in breast tumors (26)*. S. pyogenes* also correlated with *GUSBP4*, *GUSBP9*, and *GPA2* expression levels, implicating the microbe in the glucuronidation of estrogen and, indirectly, potential cell-cycle progression due to upregulation of estrogen (26).

This research suggests similar findings to the microbiome’s influence in other facets of health. There are certain bacteria that appear pathogenic in breast cancer, supporting breast tumor growth and progression, and there are those which appear to protect against breast tumor development.

### Breast tissue microbes and host DNA damage

Urbaniak et al. were among the first to report a distinct breast tissue microbiome and to distinguish the microbiome compositionally between healthy women and women with breast cancer (3). However, this study also reports the ability of mammary microbiome species, *E. coli* and *Staphylococcus epidermidis*, isolated from women with breast cancer, to induce DNA-double stranded breaks in Henrietta Lacks (HeLa) cells, alluding to another potential pathogenic mechanism (3). Microbial interference in host genetic material may lead to the later dysregulation of host cell cycles and, eventually, the development of breast cancer. Next we will explore the current research linking the mammary microbiome to host immune modulation.

### Breast tissue microbes and host immune modulation

Importantly, the largest study to date looking at the human mammary microbiome utilized 16S rRNA gene sequencing to characterize the microbiome of human breast tissue, studying a total of 221 patients with breast cancer, 18 individuals predisposed to breast cancer, referring to individuals with a genetic predisposition *via* a pathogenic gene carrier, first-degree relative with breast cancer, or past personal history of breast cancer, and 69 controls (8). This group identified decreased alpha diversity and altered microbiota composition in tumor and high risk tissues relative to controls and adjacent normal tissues (8). Expanding beyond compositional analysis, this study also developed microbiome-immune networks, correlating microbial profiles with host immune cell populations of different breast tissues, e.g., from healthy women, women at high risk of breast cancer, and women with active breast cancer (8). They observed a more disconnected immune-microbiome network structure in tumor tissues compared to benign tissue (healthy control and high risk tissues) (8). They also reported that tumor-depleted taxa, *Streptococcus* and *Propionibacterium*, were positively correlated with T-cell activation-related genes (8). This study suggests possible tumor-suppression *via* immune modulation and supports analysis of the breast tissue microbiome as a biomarker of breast cancer risk. In the next section, we explore further the bidirectional relationship between host and microbes, discussing the association of breast cancer with altered microbiome metabolic capacity.

### Metabolic alterations in the breast tissue microbiome associated with tumor development

Recent studies have investigated the metabolic function of the breast tissue microbiome using Phylogenetic Investigation of Communities by Reconstruction of Unobserved States (PICRUSt or PICRUSt2, most recent version) (27) These studies have identified a diverse array of metabolic contributions and responses of the microbiome in breast cancer. For example, one group conducted 16S rRNA sequencing on aseptically collected breast tissue from women with benign (n = 13) or malignant breast tumors (n = 15), identifying the breast tissue microbiome as distinguishable from skin or buccal cells and defining key bacterial genera, namely, *Fusobacterium, Atopobium, Gluconacetobacter, Hydrogenophaga* and *Lactobacillus*, in malignant cancerous tissue (28). This group also used PICRUSt to elucidate a decrease in inositol phosphate microbial metabolism in malignant cancer samples (28). Further, they identified a decrease in cysteine and methionine metabolism, glycosyltransferase, fatty acid biosynthesis, and C5-branched dibasic acid metabolism from the microbiome in these tissues (28). This is especially relevant to breast cancer as studies have identified cysteine and methionine as important and targetable players in cancer biology and glycosyltransferase gene profiles as prognostic biomarkers (29–31).

One group identified functional changes in Kyoto Encyclopedia of Genes and Genomes (KEGG) pathways, also inferred by PICRUSt from 16S rRNA gene amplicon sequencing, in the breast microbiome of 22 benign and 72 malignant breast cancer patients aseptically collected using a needle biopsy (32). Their analysis of the breast tissue functional bacteriome in relation to breast cancer showed upregulation of glycerophospholipid biosynthesis and ribosome biosynthesis processes (32). Conversely, flavonoid biosynthesis decreased as breast cancer grade worsened (32).

Notably, our research group identified a unique breast tissue bacterial compositional signature in pre-diagnostic tissue (n = 15), healthy tissue collected before the women were diagnosed with breast cancer, relative to healthy controls (n = 50), normal adjacent tissue (n = 49), and tumor tissue (n = 46) (7). This signature was enhanced in tumor and adjacent normal tissues (n = 49 and n = 46, respectively), suggesting bacterial dysbiosis as an early event in breast tumor development (7). We applied PICRUSt2 on the 16S rRNA amplicon sequences from these tissues and observed significant metabolic dysregulation in tissues from women diagnosed with breast cancer (pre-diagnostic, tumor, and adjacent normal), suggesting Warburg metabolism of the microbiome and a potential response of the breast tissue microbiome to a changing microenvironment during breast tumor development (7). Further, integral within the host-microbiota interactions discussed previously, we conducted preliminary correlation analyses between host transcriptome profiling and the microbial taxa and functionally-annotated genes in healthy and prediagnostic tissues (7). We identified altered associations between the host transcriptome and mammary bacterial taxa and functional bacterial KEGG orthologs in prediagnostic tissue compared with healthy tissue (7).

Characterization of the metabolic output of the bacteriome using tools such as PICRUSt and PICRUSt2 are resourceful methods to begin establishing breast tissue host-microbiome interactions in breast cancer (27). This may be expanded upon in future studies using shotgun metagenomic sequencing to evaluate the genes within the tissue as compared to those that are predicted to be present. Regardless, these studies implicate microbial functional dysbiosis in breast cancer, with most recent studies suggesting a bacterial response to the changing tumor microenvironment.

Altogether, the current literature suggests that breast tissue microbial compositional and functional dysbiosis are associated with and may be early events of breast cancer (4–7, 17, 32). Further, certain bacterial taxa act as pathogenic (e.g. *Staphylococcus epidermidis*, *Ralstonia* spp) (17, 18) or probiotic organisms (e.g. *Lactobacillus*) in breast tumor development (7, 17). The literature also suggests that these pathogenic or probiotic species can interact with the host in a variety of contexts; modulating the immune system to prevent tumor growth or enhancing cell-cycle progression, promoting tumor growth (8, 24–26). However, more research in these areas is necessary to corroborate these findings and identify potential bacterial species that could be used as microbial therapeutics or biomarkers for this disease. There is also much to be elucidated regarding host-microbiome interactions in the local breast tissue microenvironment that dictate breast tumor initiation. Future studies should also seek to elucidate the means by which bacteria identified in population-level studies alter or are altered by the formation of breast tumors in patients by coupling these clinical studies with translational animal models that can establish mechanisms. Additionally, it will be prudent to also consider the microbiomes of other body sites as they relate to breast cancer. The next section of this review focuses on the gut-breast cancer axis, highlighting studies key in linking the gut microbiome to breast cancer development.

### The gut-breast cancer axis

Another facet of breast cancer microbiome research involves gut bacteria. The importance of this microbial consortium in human health has long since been established (33). In the next few sections, we outline the current perspectives surrounding the role of the gut microbiome in breast cancer, highlighting studies that characterize the gut-breast axis in both mice and humans, and the roles of gut-derived microbial metabolites and the estrobolome in breast cancer risk (**Table S2**). Though there is mounting evidence that gut bacteria can and do influence breast tumor development, which bacteria are the most influential and the mechanisms by which they promote or prevent tumor initiation are still under investigation.

### Human studies of the gut microbiome in breast cancer

Clinical studies applying next-generation sequencing and other genomics strategies are key in identifying microbial candidates for translational animal models and defining associations between common risk factors of breast cancer and gut microbial dysbiosis. Luu et al. applied 16S rRNA sequencing together with qPCR and characterized fecal samples from breast cancer patients in various stages of the disease (34). They observed differences in *C. coccoides, F. prausnitzii*, and *Blautia*, short-chain fatty acid (SCFA)-producing bacteria that have been associated with beneficial health outcomes (35, 36), according to the clinical stages of breast cancer and the histo-prognostic grades of corresponding donors (34). Zhu et al. reported on an analysis of the gut microbiomes of 18 premenopausal breast cancer patients, 25 premenopausal healthy controls, 44 postmenopausal breast cancer patients, and 46 postmenopausal healthy controls through shotgun metagenomics (37). They observed higher microbial diversity within breast cancer patients and 45 differential species between postmenopausal patients and controls, e.g., 38 species, including *Escherichia coli*, were enriched in breast cancer patients while 7, including *Eubacterium* and *Lactobacillus* spp., were depleted (37). They also noted changes in the functional genetic potential within breast cancer patients typified in the enrichment of lipopolysaccharide (LPS) biosynthesis, iron complex transport system, phosphotransferase system, secretion system, and beta-oxidation genes (37).

In contrast to this study, another group observed that the Shannon diversity index of the gut microbiome was lower in women with breast cancer as compared to healthy controls (38). This difference may be mediated by a relative enrichment in Firmicutes, as well as a depletion in Bacteroidetes in patients diagnosed with early breast cancer compared to that of healthy women (38). The malignant or benign nature of breast tumors may also be interrelated with the gut microbiome, as Yang et al. have initially begun to explore in a 16S rRNA-sequenced gut microbiome pilot study of 83 women with invasive ductal breast carcinoma and 19 women with benign breast tumors (39). They observed no differences in diversity metrics between the groups but did note significant dysregulation of metabolic pathways in the malignant tumor group, identified *via* PICRUSt (39). Further, a recent study of the gut microbiome, characterized by 16S rRNA sequencing, of 26 subjects with breast cancer, 20 with benign breast lesions, and 20 matched healthy controls by Ma et al. reported that breast cancer patients had significantly lower alpha diversity indices, with alterations in species, such as higher levels of *Porphyromonas* and *Peptoniphilus* spp., in breast cancer patients (40).

Observations of the naturally occurring human gut may elucidate its impact on or change following the development of breast cancer, but there are various perturbations within the gut which have also warranted investigation. For example, the type of breast tumor diagnosed can shape clinical prognosis, provide essential insight into how a patient will be impacted by breast cancer, and determine the mode of treatment (41). Depending on the stage of life patients experience breast cancer, the hormonal levels within the body and gut may also be variable and influence health (42) and microbiota statuses (43). Many patients also require surgical removal of tumors, which is typically accompanied by prophylactic antibiotics and/or chemotherapy, both of which can instigate and or exacerbate microbial compositional alterations (6, 44, 45); we explore these perturbations later in this review. In the next sections, we focus on animal model studies of the gut-breast cancer axis, wherein the mechanisms by which specific pathogenic or probiotic bacteria may influence or inhibit tumor development may be elucidated.

### Mouse models to study gut microbes in breast cancer

Similar to animal and cell-based studies of the local breast tissue microbiome, animal model studies of the gut microbiota in relation to breast cancer primarily focus on specific bacterial taxa and metabolites present within the gut and how these taxa correlate with either preventive or pathogenic effects (46–50). A number of these studies highlight the immune-modulatory effects of specific bacteria (47–50), characterizing these microbes as potential probiotics to combat breast cancer.

### Characterizing gut pathogens in breast cancer

Lakritz et al. tested a mechanistic hypothesis of gut microbiota-modulation of distal breast cancer using *Helicobacter hepaticus*, a known pathogen (51), in a microbial infection animal model (46). Following orogastric infection of mice with *Helicobacter hepaticus*, researchers observed subsequent infiltration of myeloperoxidase-positive neutrophils in the mammary tissue and tumorigenesis (46). Tumorigenesis was inhibited by the depletion of these neutrophils, alluding to the important role of intestinal microbial balance in distal tumor development and the cancer promoting inflammatory response (46).

### Probiotic gut microbes protect against breast tumor progression

Conversely, certain probiotic bacteria, such as *Lactobacillus* spp., have been heavily studied in mouse models and the immunomodulatory capabilities of these species further elucidated in breast cancer. One study used FVB strain erbB2 (HER2) mutant mice with genetic prepositions to mammary tumors and found that when supplied with *Lactobacillus reuteri* to the gut, the mouse immune system triggered CD4^+^CD45RBloCD25^+^ lymphocyte (Treg cells) protective mechanisms to inhibit cancer progression (47).

Further, oral administration of *L. acidophilus* to Balb/C female mice was found to induce a decrease in breast tumor growth patterns and altered production of interferon (IFN)-γ, interleukin (IL)-4, transforming growth factor (TGF)-β, and lymphocyte proliferation, favoring antitumor immunity and reducing tumor growth (52). Dallal et al. studied the effects of oral administration of *L. casei* on natural killer cell cytotoxicity and production of cytokines in the spleen cell culture of BALB/c mice with concurrent invasive ductal carcinoma (n = 30 female in-bred BALB/c mice, divided into two groups of test and control each containing 15 mice) (48). Their results showed that oral administration of *L. casei* increased the production of IL-12 and IFN-γ, increased natural killer cell cytotoxicity in spleen cell culture, prolonged survival, and decreased the growth rate of tumors in the test mice (48). Building upon this, Yazdi et al. reported that *Lactobacillus brevis* treatment is more effective when the bacteria is combined with biogenic immunomodulating selenium nanoparticles (SeNPs) (n = 60 female inbred BALB/c mice, divided into 4 groups–either given oral PBS daily and injected by this buffer after tumor induction (control), given 100 μg/day of SeNPs as an oral supplement for 30 days, given no supplementation of SeNPs and injected with 4T1 cell crude antigens, or supplemented 100 μg/day SeNPs for 30 days and simultaneously injected with the crude antigens–and each n = 15 mice) (49). The authors identified increased levels of IFN-γ, IL-17, natural killer cytotoxicity, extended life span, and decrease in the tumor metastasis to the liver in SeNP-enriched *L. brevis* administered mice as compared to control mice or mice given *L. brevis* alone (49). This is most likely mediated by the SeNPs’ ability to directly interact with innate immune cells such as dendritic cells, macrophages, and natural killer cells and regulate innate immunity (53). Finally, one study focused on the antitumor effects of *Lactobacillus* by studying immune cells in mammary glands and the cytokine concentration in serum of mice fed with milk fermented by *Lactobacillus helveticus* (50). The mice were fed for 7 days prior to being injected or not with breast tumor cells, and then fed milk 4 days following injection (50). The researchers measured immunoglobulin (Ig) A, CD4, CD8, cytokines and Bcl-2 positive cells in mammary glands, and cytokines in serum (50). They found that mice fed with *L. helveticus* R389 fermented milk had a modulated immune response, including increased IgA and CD4 positive cells in mammary glands, increased anti-inflammatory IL-10, and decreased inflammatory IL-6 (associated with poor breast cancer prognostic outcomes), indicating that *L. helveticus* has a regulatory role in the breast tissue environment (50). The authors concluded that milk fermented by *L. helveticus* R389 could be used as an oral immune adjuvant to protect against mammary gland pathologies such as cancer (50). This study alludes to the use of dietary interventions, which may alter the gut microbiome composition and influence protection from or progression of breast tumors.

### Dietary influences on the gut-breast cancer axis

Murine models can also be used to incorporate a carefully controlled diet to assess its role in the gut-breast cancer axis. Ma et al. studied the effect of the combination of berberine, a chemical found in plants such as grapes, turmeric, and goldthread, and exercise as an anti-tumor treatment in mice (54). Together with a significantly slowed progression of breast cancer in 4T1 tumor-bearing mice, they observed significantly increased levels of SCFAs, microbial-derived metabolites, in mouse gastrointestinal tracts, which may influence general inflammation (54).

Lakritz et al. used two different mouse models to analyze the development of mammary cancer when eating a Westernized diet as compared to a genetic predilection to breast cancer, with the additional variable of *Lactobacillus* supplementation (47). Mammary carcinogenesis was inhibited by routine exposure to *Lactobacillus reuteri* ATCC-PTA-6475 in drinking water in the Westernized diet model (47). The second model (FVB strain erbB2 (HER2) mutant mice, genetically susceptible to mammary tumors fed regular chow) showed that oral supplementation with *L. reuteri* was also sufficient to inhibit features of mammary neoplasia (47). Specifically in relation to the development of a breast tumor itself, researchers determined the protective mechanism to be microbially-triggered CD4^+^CD25^+^ lymphocytes; the transplantation of these cells to other murine subjects generated similar results for the inhibition of mammary neoplasia and tumors (47). Finally, Zamberi et al. studied the effects of kefir, a cultured product containing probiotics, made from kefir grains cultured in Malaysia as an anti-inflammatory, immunomodulatory, and anticancer treatment (55). They first treated 4T1 cancer cells with kefir water *in vitro* to assess its effects; they then injected BALB/c mice with the cancer cells and treated them orally with kefir water for 28 days (55). Remarkably, the kefir water was cytotoxic toward the cancer cells at half-maximal inhibitory concentration of 12.5 and 8.33 mg/mL and a reduction in tumor size, reduction in weight, and a substantial increase in helper T cells and cytotoxic T cells were observed in the kefir water-treated group (55)

The animal studies described above highlight probiotic and diet-induced host immune responses, which are key in combating and preventing breast tumor growth and proliferation. These represent critical pathways to explore further in clinical trials of the microbiome and breast cancer.

### Pending clinical trials in the gut-breast cancer axis

A significant step forward in gut microbiome-breast cancer studies in humans is the establishment of clinical trials to assess whether the modulatory effects of certain taxa discussed in the previous sections can influence breast cancer development/progression. Recent studies include one by the Mayo Clinic in Jacksonville, Florida (recently completed in March 2023, results pending), evaluating whether engineering the gut microbiome *via* probiotics, and thereby facilitating a diverse microbiota population, might shape the immune system’s reaction to operable stage I-III breast or lung cancers (56). Another prospective study is still underway by Hackensack Meridian Health, Yale University, and Georgetown University (57). This is a study of newly diagnosed triple negative breast cancer patients undergoing neoadjuvant chemotherapy (57). Researchers are aiming to correlate gut and intratumoral microbiome composition and anti-tumor immune responses (57). Finally, clinical trials testing whether probiotics, containing bacteria such as *Lactobacillus*, will beneficially affect the immune system during the course of breast cancer have been carried out (58, 59), with pending results. It is exciting that research connecting current translational mouse model studies with human cohort studies is already underway. In the next section, we will discuss additional pathways within the gut-breast cancer axis, the relationship of gut microbial metabolites with breast tumor development and the estrobolome’s influence on breast cancer. Both pathways are relatively underexplored, highlighting additional research areas within this field that may yield key insights in breast cancer development and progression.

### Gut microbial metabolites in breast tumor development

The gut microbiome is responsible for numerous metabolites utilized by our host cells as energy sources and immune-modulators. Literature suggests bacterial metabolites, such as bile acids, SCFAs, and cadaverine, as influential in inhibiting breast tumor development and progression.

Studies report the association of lithocholic acid (a bacterial derived bile acid) with *Clostridiales* spp. and its ability to reduce breast cancer proliferation and vascular endothelial growth factors (60–63). SCFA receptors are also reported to inhibit invasive phenotypes in breast cancer cells *via* the activation of cognate receptors (64). Sodium butyrate in particular facilitates histone deacetylase inhibition, modulation of glycolytic pathways, pyruvate kinase, lactate dehydrogenase, oxygen-consuming activity, and apoptosis in breast cancer cells through reactive oxygen species (ROS) formation and mitochondrial impairment (65, 66). Similarly, researchers report that SCFA-producing *E. coli* strains, namely KUB-36 which is a non-exotoxin producer, exhibit simultaneous cytotoxic and anti-inflammatory effects on cancer cells (67).

Indolepropionic acid (IPA) exhibits cytostatic properties and selectively targets breast cancer cells while having no effect on non-transformed, primary fibroblasts (68). IPA reduced the proportion, proliferation, and metastasis of cancer cells by inducing epithelial-to-mesenchymal transition and oxidative stress and facilitated anti-tumor immune activity (68). Akin to the mechanism of IPA, bacteria use diamines, in this case, cadaverine, to buffer the pH of their environment, and cadaverine has been seen to reverse endothelial-to-mesenchymal transition and inhibit cellular movement and invasion in breast cancer cell lines (63).

Altogether, these bacterial metabolites may play early and continuous regulatory roles in the progression of breast tumors. Yet, the origin of many of these metabolites have yet to be fully described or mechanistically interrogated due to the inherently complex interactions between members of the gut microbiota and between the microbiota and human body. Regardless, they represent a key mechanistic connection between the gut microbiome and breast cancer, which should be expanded on in further research.

### The estrobolome, estrogen, and breast cancer

One final area to be discussed in relation to the gut-breast cancer axis is the gut estrobolome and its potential influence on estrogen regulation as it relates to breast cancer. The functional gut estrobolome is defined as a collection of bacterial genes encoding enzymes essential in estrogen metabolism, which aids in modulating the enterohepatic circulation of estrogens (43). Endogenous estrogens are critically related to the risk of breast cancer in postmenopausal women (69, 70). The hormone receptor–positive (HR+)/HER2–negative subtype is also the most common subtype of breast cancer found in post-menopausal women (71, 72). The activation of estrogen receptors in breast tissues often causes an increase of cells entering the G0 and G1 phases and cell proliferation that is commonly seen in breast cancer patients (73). Thus, in connection with the alterations in bacterial populations seen within breast cancer, host hormonal metabolism changes seen during breast cancer may be due to the shifts in host gut estrobolome diversity and abundance, affecting overall levels of endogenous estrogen (74, 75).

The estrobolome is able to regulate endogenous estrogen through many pathways. One possible pathway is through the abundance modulation of bacterial species that produce β-glucuronidases (GUS) and β-glucuronides (43, 76). Ervin et al. conducted an *in vitro* study of gut microbial GUS enzymes and found that GUS can reactivate the inactivated estrogen form of estrone-3-glucuronide and estradiol-17-glucuronide to estrone and estradiol (77). The menopause-associated estrobolome dysbiosis in favor of bacterial species that produced GUS likely contributes to a higher estrogen load on the host and, thus, a greater risk for carcinogenesis (69, 70, 77). The estrobolome may also synthesize estrogen-like compounds through the breakdown of normally indigestible dietary fibers and polyphenols that exhibit varied estrogenic potency, contributing to the host’s estrogen load and breast cancer carcinogenesis to varying degrees (75). Little research relating the estrobolome to breast cancer has been conducted and this represents an area of research within the gut-breast cancer axis that begs further exploration. As risk factors such as age at menopause and obesity are closely linked to estrogenic shifts and the gut microbiome, our next section highlights the potential role of the microbiome as a mediator between these risk factors in breast cancer.

## The influence of breast cancer risk factors and therapeutic exposures on the microbiome

Breast cancer is a multifactorial disease, influenced by many environmental and genetic risk factors (78). Significant research has been conducted on the effects of therapeutic exposures, such as prophylactic antibiotics and chemotherapies, on the microbiome in relation to breast cancer. Since the microbiome represents a key component of the tumor microenvironment as well as a potential causal influence on breast tumor development and progression, assessment of this microbial consortia in cancer therapeutic strategies is critical to holistically treat patients with this disease.

### Breast cancer clinical risk factors: age, menopause, and obesity, and the microbiome

Studies have shown that later age at menopause is significant in the development of breast cancer (79, 80). Intricately linked with this connection is age; it is an important factor in extreme variability and phylum proportions in the gut microbiome, potentially due to factors such as lowered nutrition availability to the gut (80–82). Goedert et al. observed decreased diversity at the community level of the gut microbiome using 16S rRNA sequencing data of microbial DNA present in the fecal samples of 48 postmenopausal breast cancer patients as compared to 48 control patients (83). This decrease in diversity was independent of total estrogens, which correlated with α-diversity in control patients (Spearman Rho = 0.37, P = 0.009) but not case patients (Spearman Rho = 0.04, P = 0.77) (83). For premenopausal women, one research group notes a reduction in gut microbiome α-diversity and alterations in β-diversity in breast cancer patients (80). The study reports 14 microbial markers identified in the different menopausal statuses of breast cancer, including *Bacteroides fragilis* in young women of premenopausal statuses and *Klebsiella pneumoniae* in older women of postmenopausal statuses (80). Zhu et al. studied 18 premenopausal breast cancer patients, 25 premenopausal healthy controls, 44 postmenopausal breast cancer patients, and 46 postmenopausal healthy controls: they found that the relative abundance of 45 species differed between postmenopausal patients and postmenopausal controls: 38 species were enriched in postmenopausal patients including a number of gram-negative species, and 7 species were less abundant in postmenopausal patients, including gram-positive *Eubacterium eligens* and *Lactobacillus vaginalis* (37).

Obesity, which involves low-grade inflammation related to gut dysbiosis, is another factor implicated within the development of breast cancer (84, 85). A systematic review of 12,496 studies up until August 2019 revealed that obese individuals have a greater Firmicutes/Bacteroidetes ratio, as well as less Verrucomicrobia (*Akkermansia muciniphila*), *Faecalibacterium prausnitzii*, *Bacteroidetes*, *Methanobrevibacter smithii*, and *Lactobacillus plantarum* and *paracasei* (86). They concluded that individuals with obesity showed gut microbiota profiles different from lean individuals (86). Houssain et al. conducted a metagenomic study of a syngeneic mouse model of triple-negative breast cancer (87). They found that obesity significantly lowers the alpha diversity of the gut microbiome in this mouse model and that tumor progression was significantly accelerated in obese mice compared to controls, suggesting a relationship between obesity-associated gut dysbiosis and breast cancer progression (87). However, more comprehensive studies are needed to establish a mechanistic relationship between breast cancer and obesity itself.

The risk factors of later age at menopause and obesity clearly affect the human microbiome, but the nuances of these effects need clarification through larger and more technologically extensive studies. Further, it is not yet clear the effects of these risk factors on the local breast tissue microbiome. The presence of certain microbiota alter health outcomes, but their utilization of available resources, metabolic output, and interaction with host cells and other bacterial organisms may provide insight into their role in breast cancer development. Thus, studies exploring the effect of co-morbidities upon differences in microbiota presence, their function, and metabolic output, in combination with breast cancer diagnoses and outcomes, will be essential in the progression and expansion of the field as a whole.

### Antibiotic-induced differences in the microbiome of breast cancer patients

Antibiotics may positively or negatively alter the gut microbiome composition in breast cancer patients (44, 88, 89). Prophylactic antibiotics are often prescribed for breast-cancer associated surgeries (90). But population-based analyses of antibiotic usage in female breast cancer patients report modest positive associations between antibiotic usage and breast cancer development (90, 91). These antibiotics are orally administered and have the potential to directly affect the bacterial populations within the body, which then may affect bacterial modulation of immune and tumor cell development and subsequent cancer treatments (91, 92).

Antibiotic-treated mice injected with tumor cells are also reported to respond poorly to CpG-oligonucleotide treatment and chemotherapy: such mice lack a balanced commensal gut microbiome, have depleted myeloid cell counts, and are less responsive to oxaliplatin, a chemotherapeutic, for tumor regression (45). Notably, McKee et al. report that antibiotic-induced microbiota disturbances promote tumor growth in several breast cancer models, that increased tumor volume positively correlates with stromal mast cell density, and that supplementation with *Faecalibacterium rodentium* restores tumor growth to normal (93).

An interesting study by Rosean et al. tested how pre-cancer development of dysbiosis within the gut microbiome affects breast cancer (6). They found that the pre-established disruption of commensal microbiota homeostasis *via* antibiotic gavage (confirmed using 16S rRNA sequencing) resulted in enhanced circulating tumor cells, increased fibrosis and collagen deposition both systemically and locally within the tumor microenvironment, and significant myeloid infiltration into the mammary gland and breast tumor (6).

Interestingly, antibiotic exposure contributes to gut dysbiosis, which is reported to enhance tumor progression and worsen patient responses to cancer therapeutics. Further, per the study by Rosean et. al., antibiotic induced gut dysbiosis may be key in initiation of breast cancer (6). Though prophylactic antibiotics are often necessary prior to surgery, these studies emphasize the need to approach antibiotic exposure as it relates to breast cancer with caution. In the next section, we will discuss the microbiome in cancer therapies, highlighting a fragile relationship between our microbiome and our body’s response to cancer therapeutics.

### Breast cancer chemotherapy and modulation of the microbiome

The microbiome of cancer patients is also affected by chemotherapeutics. A recent study of shotgun metagenomic sequencing of 121 stool samples from 76 breast cancer patients (n = 45 for samples prior to and post-chemotherapy) by Terrisse et al. found that an overabundance of microbiota commensals may negatively influence the outcome and side effects of breast cancer treatments and validated these findings in murine models of breast cancer (94). Similarly, Aarnoutse et al. recently observed changes within the intestinal microbiota composition of 16S rRNA-sequenced stool samples from 44 patients undergoing chemotherapy, with the abundance of Proteobacteria, unclassified Enterobacterales, *Lactobacillus*, Ruminococcaceae NK4A214 group, Marvinbryantia, Christensenellaceae R7 group, and Ruminococcaceae UCG-005 changing significantly over time and decreased species richness when patients were affected with diarrhea (95). Moreover, in a 4T1 mammary carcinoma experimental mouse model and using 16S rRNA sequencing, researchers were able to investigate the effects of Vismodegib treatment on the gut microbiota (96). The researchers observed remodeling of the gut microbiota and an increase in proliferative CD8+ T cells in the colonic immune network (96).

In some cases, chemotherapies have been reported to instigate tumor-progressing microbiota, while in others, probiotic microbes can enhance tumor-targeting immune responses. Chiba et al. evaluated the effect of neoadjuvant chemotherapy on the human tumor microbiome (97). They used snap-frozen aseptically collected breast tumor tissue from women who underwent neoadjuvant chemotherapy (n = 15) or women with no prior therapy at the time of surgery (n = 18) and performed 16S rRNA sequencing to identify tumor bacterial populations (confirmed with staining of breast tumor microarrays) (97). They found that chemotherapy administration increased *Pseudomonas* spp. in breast tumors (97). Further, in patients that experience metastases, breast tumors showed increased *Brevundimonas* and *Staphylococcus* (97). Finally, they display the direct tumor modulating ability of bacteria *via* inoculation of tumor cells with *Pseudomonas aeruginosa* supplemented media and show how this bacterium can modulate doxorubicin (Dox) (chemotherapeutic)-mediated cell death, either enhancing or inhibiting the drug’s effect based upon the cell line under study (97). Metabolites that had consistent effects included LPS, which stimulated cancer cell proliferation, and pyocyanin (a *P. aeruginosa*-derived metabolite), which potentiated Dox effects and cancer cell death (97). Viaud et al. demonstrated that cyclophosphamide, a class of medications called alkylating agents that slows or stops the growth of cancer cells and suppresses the immune system, alters small intestine microbiota composition in mice, and induces the translocation of specific Gram-positive bacteria into secondary lymphoid organs (98). These bacteria then stimulate the generation of a specific subset of “pathogenic” T helper 17 (pTH17) cells and memory TH1 immune responses, thereby augmenting anti-tumor activity (98). Although antibiotics could disrupt the effectiveness of cyclophosphamide, its efficacy could be restored by *Enterococcus hirae* and *Barnesiella intestinihominis* (98).

Finally, Bawaneh et al. recently performed a similar analysis utilizing Dox, an anthracycline treatment that interrupts cancer cell growth by blocking topoisomerase 2, in female BALB/c mice (n = 115) injected with 4T1 cancer cells (99). The researchers treated these mice with Dox in combination with antibiotics, diet-derived fecal microbiota transplantation (FMT), and/or exogenous LPS (these groups were then stratified into Dox responders or Dox nonresponders) (99). One of the most notable results from this experiment was that Dox responders and groups treated with antibiotics in addition to Dox displayed reduced tumor weight and metastatic burden (99). Further, the researchers found that Dox was associated with increased *Akkermansia muciniphila* (99), a well-studied mucin-degrading bacterium that has been inversely associated with many adverse health outcomes (100). In contrast, when mice were treated with Dox in combination with a high-fat diet-derived FMT, tumor growth continued and Dox was not as effective (99). The researchers found that these reduced Dox effects were interrelated with microbial components of both the gut and plasma through the increase in LPS (99). When LPS was injected in its exogenous form, this was followed by intestinal inflammation, reduced Dox responsiveness, and increased lung metastasis in Dox nonresponding mice and those that were treated with Dox and an FMT (99).

These studies highlight, again, opposing effects of specific bacteria in breast tumor progression and the potential to target specific microbes as breast cancer therapeutics. Further, it is clear that microbiome interaction with host immune functions is critical to the host’s response to chemotherapeutics. In the future, it will be critical to conduct clinical trials that assess the effect of breast-cancer probiotic microbes before and after chemotherapy. These studies should prepare to analyze host immunological responses following probiotic treatment to further elucidate if the microbiome mediates tumor-targeting immunity when undergoing chemotherapy.

## Discussion

**Figure 1:**
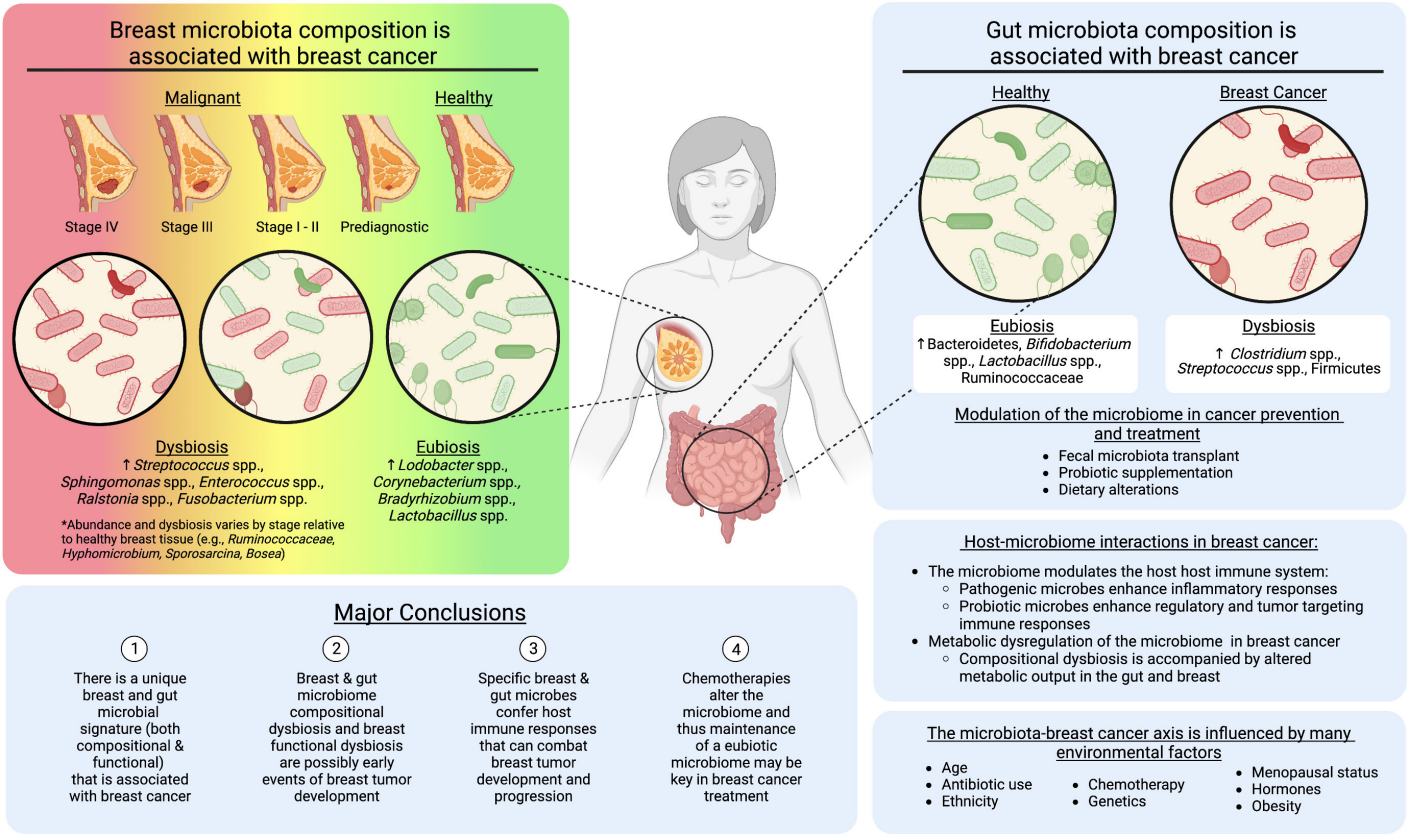
The role of the breast and gut microbiomes in breast cancer development. Created with BioRender.com.

In this review, we outline studies analyzing the breast and gut microbiomes in breast cancer initiation and progression, and during chemotherapy. From the literature, we identified the following major conclusions, (I) There are unique breast and gut microbial signatures (both compositional and functional) that are associated with breast cancer, (II) breast and gut microbiome compositional and breast functional dysbiosis represent potential early events of breast tumor development, (III) specific breast and gut microbes confer host immune responses that can combat breast tumor development and progression, and (IV) chemotherapies alter the microbiome and thus maintenance of a eubiotic microbiome may be key in breast cancer treatment (**Figure 1**).

The current research suggests an intricate, bidirectional relationship between the gut and breast microbiomes and breast cancer development. Based on our assessment of the current literature, there is evidence that the breast tissue and gut microbiome composition is altered following breast cancer diagnosis (4–7, 15, 17, 37, 46–50, 61). However, recent work suggests a progression toward enhanced dysbiosis as the breast tumor tissue progresses from early stages, prior to diagnosis, through later stages of breast cancer (6–8, 17) **(** **Figure 1**). These compositional shifts are also accompanied by changes in the metabolic output of the microbiome (7, 28, 32, 39). Metabolic dysregulation may be a by-product of compositional dysbiosis, due to the loss of specific bacterial taxa. However, it also suggests the microbiome is in functional disarray due to the changing microenvironment, which along with histological and transcriptomic abnormalities present in pre-diagnostic tissue (101), may be another early event of breast tumor initiation. For example, Marino et al. used microdissection and whole-transcriptome profiling of human breast epithelium prior to and post tumor diagnosis and revealed significant upregulation of genes involved in lipid metabolism, including fatty acid uptake/transport, lipolysis, and lipid peroxidation, which may create a more favorable environment for cancer cell transformation, proliferation, and survival (101). Our recent manuscript characterized the microbiome of pre-diagnostic tissue from some of the same women, highlighting a decreased abundance of microbial lipid metabolism genes, suggesting a response of the microbiome to the changing tumor microenvironment (7). A holistic understanding of the key events that establish and progress breast cancer will be important for the development of cancer therapeutics. However these works also highlight the potential to use bacteria and bacterial metabolites as biomarkers for this disease. Notably, the current work on the pre-diagnostic microbiome is specific to the local breast tissue bacteria (7, 8). It will be critical in future studies to include the breast and gut microbiomes in analyses of the breast tumor microenvironment before and during tumor initiation. This should involve multi-omic strategies within pre-diagnostic tissues, and translational animal models, wherein the microbiome is disrupted prior to breast tumor initiation.

We also determined through this literature review that specific bacterial taxa in the breast and gut are reported as pathogenic, enhancing tumor progression (e.g. *Ralstonia* spp., *Streptococcus epidermidis*), or probiotic, stalling tumor progression (e.g. *Lactobacillus* spp.*, Corynebacterium* spp.) (3, 17, 18, 49, 50). Additionally, though the local and gut microbiomes interact with the host in a variety of ways, the current literature suggests that host immune-modulation is the most influential pathway by which microbes influence breast tumor progression or inhibition (8, 46–49). The majority of studies where immune factors have been assessed involve the gut microbiome (46–49), and thus there is a need to expand breast tissue microbiome studies to include host immune characteristics. Reported evidence that specific microbial taxa can be both pathogenic and probiotic in breast cancer is also highly related to the immune response elicited by the host (46–49). Specific taxa are pathogenic and instigate a response that can progress tumor development, while others act as probiotics, supporting regulatory or anti-inflammatory immune responses that slow or even stall tumor progression (46–49). These findings are mirrored in studies of chemotherapeutics that include analysis of the microbiome, suggesting that specific taxa are not only pathogenic, supporting cancer progression, but they also may stall treatment of breast cancer (98–100). Consequently, future studies analyzing host-microbiome interactions in breast cancer should focus on host immune markers among pre-diagnostic and post-diagnostic cases, which could be further divided into chemo-treated and untreated groups.

Aside from these avenues for further research in this field, we also note that there are highly underexplored research areas that will help bridge our understanding of host-microbiome interactions in breast cancer. Firstly, the role of the estrobolome in breast cancer has not been clearly elucidated. This will require multi-omics strategies and, potentially, a targeted or hypothesis driven approach and re-exploration of functional metagenomic data from the breast and/or gut microbiomes. Further, metabolomics is a natural addition to dietary intervention studies in breast cancer. Targeted and untargeted metabolomics in conjunction with microbiome and host-genome/transcriptome sequencing will be key in establishing additional mechanistic links between the local and gut microbiomes and breast tumor development. Lastly, analysis of breast cancer subtypes, stages, and effects on the microbiome stimulated by factors such as obesity, menopause, and age, should be further explored. This will require large cohort studies where appropriate statistical power can be maintained even after establishment of sub-groups.

Altogether, the current literature highlights the potential to identify probiotic bacterial taxa that could be used as breast cancer therapeutics or shifts in bacterial abundances that could be used as biomarkers of breast cancer. It is evident that certain bacteria within the gut and breast microbiomes do play roles in cancer pathogenicity, while others serve as probiotics, supporting host health and protecting against breast cancer. However, it is equally apparent, based on the current literature, that both of these bacterial consortia may be responding to breast cancer. The relationship between host and microbiome in breast cancer is bidirectional, but clarification of the bacterial taxa that fall into these two categories is necessary to develop microbe-based treatment strategies and clarify how response of the microbiome to cancer and cancer therapeutics might inhibit cancer treatment.

### The future of the microbiome in breast cancer prevention and treatment

As we look toward future perspectives on the utilization of microbiome discoveries for the prevention and therapeutic treatment of breast cancer, the outlook for microbe-based therapeutic strategies to prevent or treat breast cancer is promising. Analysis of the breast tissue microbiome offers potential for predictive biomarkers, but more research is needed to determine how this microbial consortium is seeded and maintained before modulation of this microbiome can be applied as a breast cancer treatment. Alteration of the gut microbiota, however, shows promise for microbiota modulation in relation to breast cancer. Probiotic and prebiotic supplementation methods are attractive prospects, particularly as these supplementations are shown to induce immune modulations that protect against breast tumor development (49, 52). However, the persistent presence of these supplemented bacteria has not been demonstrated (102). Future clinical trials (discussed in this review) will be key in clarifying the effects of beneficial bacterial species in the prevention and treatment of breast cancer (56, 57). However, the wholesale transfer of a eubiotic microbiota *via* fecal microbiota transplant (FMT) from healthy individuals to those at high risk of developing, or those that have developed, cancer represents another avenue to harness the gut microbiome as a breast cancer therapeutic (103–105).

FMT has most successfully been applied in humans for the treatment of *Clostridium difficile* infections following the treatment of antibiotics (106). It has also been demonstrated as an effective cancer treatment in preliminary studies, particularly in colon cancer and potentially in melanoma skin cancers (103, 107, 108). Further, recent work presented at the American Society of Clinical Oncology highlights the use of FMTs from healthy individuals to patients receiving immune checkpoint inhibitors and who had subsequently developed colitis to improve clinical phenotypes (109). This highlights the close interplay of the immune system with the microbiota, an avenue we note in this review as key to explore in future studies. For breast cancer specifically, one mouse-model study suggests FMT from healthy individuals compared with mice humanized by FMT from breast cancer patients can enhance the anti-cancer effects of chemotherapy (94). Another group performed FMT between mice fed control or high fat diets demonstrating the transfer of protumorigenic effects from HFD-diet mice to control mice after HFD fecal transplant (108). This group also reports that FMT alters composition of both the mammary and gut microbiomes, suggesting FMT as a potential method to establish eubiotic breast and gut microbial communities, which may improve treatment of or even prevent breast cancer (108).

In conclusion, though the breast tissue microbiome is the most direct route of tumor-microbe interaction in breast cancer, modulation of the gut microbiome, whether it be with probiotic supplementations, dietary changes, or FMT, may support promotion of eubiotic states in both body niches (108) (**Figure 1**). Ultimately, the interplay of various microbial components, including bacteria, colonocytes, archaea, viruses, fungi, protists, and metabolites, of an FMT and their respective roles is still under study (110), but the immunostimulatory effects, competitive exclusion, and/or prevention of a self-potentiating dysbiotic inflammatory state supports the usage of FMTs cancer therapeutics. Further, transfer of a collective eubiotic microbial community, rather than specific probiotic species, which may be outcompeted by dysbiotic taxa, has the potential to synergistically support breast cancer chemotherapies through a wider array of immune-microbe interactions (108, 109). Altogether, current research supports the microbiome as a key physiological component that cannot be ignored in relation to gut and non-gut related cancers. It may be a critical missing piece in developing holistic breast cancer treatments and preventive strategies.

## Author contributions

CH led the review outline and article collation and wrote the manuscript. RJ made the figure and tables and contributed to outlining, collating articles, and writing the manuscript. CH and RJ reviewed and edited the manuscript prior to submission. LS supervised the work and critically reviewed and edited the manuscript. All authors contributed to the article and approved the submitted version.
