# Supplementary material for: Elucidating the roles of the mammary and gut microbiomes in breast cancer development
Source: Front Oncol. 2023 Aug 17;13:1198259. doi: 10.3389/fonc.2023.1198259 (PMC10470065; doi:10.3389/fonc.2023.1198259)
Supplement: Supplementary file 1 [file Table_1.docx]

**Elucidating the roles of the mammary and gut microbiomes in breast cancer development.**

Courtney Hoskinson^1^, Rachel Yutong Jiang^2^, Leah T. Stiemsma^2*^

1. Department of Microbiology & Immunology, University of British Columbia, Vancouver, Canada, V6T 1Z3
2. Natural Science Division, Pepperdine University, Malibu, CA, 90263

**Table S1:** **Key research studies focused on the breast tissue microbiome in breast cancer**

| Research Objective | Model Population | Methods | Key Findings | Reference Number |
| --- | --- | --- | --- | --- |
| To determine if the host microbiome can affect breast cancer risk | Fresh breast tissue from patients (n=71) undergoing breast surgery at St. Joseph's Hospital in London, Ontario, Canada (benign tumor (n=13), malignant tumor(n=45), and healthy tissues(n=23))  HeLa cell line | Breast tissue local microbiome was sequenced with 16S rRNA amplicon sequencing.  Beta diversity was based on UniFrac distance. Bacterial relative abundance was compared between groups.  HeLa cells were infected with various microbes and DNA damage was assessed via immunofluorescence and the histone-2AX (H2AX) phosphorylation (γ-H2AX) assay. | There are differences in microbiome profiles between adjacent normal with breast cancer tissues and healthy tissue.  Breast cancer tissue had higher relative abundance of *Bacillus, Enterobacteriaceae* and *Staphylococcus. Escherichia coli,* and *Staphylococcus epidermidis.*  The microbial profiles between normal adjacent and tumor tissues are similar.  Cells infected with *E. coli* and *S. epidermidis* were reported to induce double stranded DNA breaks. | [(3)](https://www.zotero.org/google-docs/?eq2yKp) |
| To determine local breast tissue microbiome taxonomic composition post diagnosis through cancer development. | Women breast tissues from  pre-diagnostic (n=15), tumor (n=49), and adjacent normal tissues (n=46) | Breast tissue local microbe DNA was isolated and analyzed with Illumina MiSeq paired-end sequencing of the V3-V4 region of the 16S gene.  Alpha diversity was visualized with amplicon sequence variants (aligned with SILVA database), Chao1, Observed OTUs, and the Shannon index.  Taxon abundance plots were constructed and significant ASVs were compared with LeFSe and MaAsLin2.  PICRUSt was used to predict compositional and functional dysbiosis and analyzed with LeFSe and MaAsLin2. | Intermediary bacterial taxonomic signature identified in pre-diagnostic tissues, which is characterized by shifts in *Bacillaceae, Burkholderiaceae, Corynebacteriaceae, Streptococcaceae,* and *Staphylococcaceae.* These taxa are among those were found to be more abundant in the adjacent normal and tumor tissues.  Microbiome metabolic dysregulation was observed in pre-diagnostic, adjacent normal, and tumor tissues. | [(7)](https://www.zotero.org/google-docs/?BSByMf) |
| To determine if breast cancer development is influenced by local breast microbiome and immune responses. | Human breast tissue from breast cancer patients (n=221), women predisposed to breast cancer (n=18), and controls (n=69) | Breast tissue local microbiomes were sequenced with 16S rRNA gene sequencing and characterized.  Taxonomic groups with prognostic clinicopathologic features were identified through linear models with White’s t or Kruskal–Wallis H-tests with Benjamini–Hochberg multiple testing correction.  Bacterial taxa and immunological data from NanoString gene expression and 65-plex cytokine assays were correlated by Spearman coefficient based network analysis. | There were significant differences in relative microbial abundance depending on tissue type (tumor, tumor adjacent normal, high-risk, healthy control), cancer stage, grade, histologic subtype, receptor status, lymphovascular invasion, and node-positive status.  Tumor tissues had an absence of *Anaerococcus, Caulobacter,* and *Streptococcus*, which were present in healthy tissues.  Oncogenic immune features were inversely related to abundance of *Propionibacterium* and *Staphylococcus*, which were absent in tumor tissue.  T-cell activation genes were positively associated with the abundance of  *Streptococcus* and *Propionibacterium*. | [(8)](https://www.zotero.org/google-docs/?Zg4JYC) |
| To determine taxonomic characterization of local tumor microbiome | Human tumor samples from solid tumor resections and tissue biopsies:  Tumor samples (n=1010)  Normal (control) samples (n=516) | Human tumor sample microbes were isolated and analyzed via V4 or V3-V4 16S rDNA qPCR & sequencing, tissue staining (LPS, LTA, FISH).  Estimation of bacterial load was done by using a calibration curve and heat maps.  Bacteria presence in cancer cells was further verified with correlative light and electron microscopy (CLEM).  Tumor microbiomes were characterized by multiplexed 16S rDNA sequencing protocol. Different tumor types and microbiome characters were compared with Wilcoxon rank sum test. | Firmicutes and Bacteroidetes were the most abundant phyla in most cancers but were present in different ratios.  Colorectal tumors had highest abundances of Firmicutes and Bacteroidetes.  Pancreatic tumors had the highest abundance of Proteobacteria.  non gastrointestinal (breast, lung, ovarian) tumors had the highest abundance of Actinobacteria phylum (Corynebacteriaceae and Micrococcaceae families).  Local tumor bacteria were mainly intracellular in both tumor and host immune cells.  Breast cancer tumor displayed an especially diverse and rich microbiome. | [(14)](https://www.zotero.org/google-docs/?aTeIkX) |
| To determine microbial signatures associated with breast cancer types (endocrine receptor (ER) positive, triple positive, Her2 positive and triple negative breast cancers) | The breast tissues are collected from de-identified archived samples.  - Endocrine receptor (ER) positive (n=50)  - Triple positive (n=24)  - Her2 positive (n=34)  - triple negative (n=40)  - Control (n=20) | Human breast tissue was analyzed via whole genome and transcriptome amplification and a pan-pathogen microarray (PathoChip) strategy. This was later validated by PCR and Sanger sequencing.  Microbial signatures were detected by Hierarchical cluster analysis. | There were distinct microbial signatures for the triple negative and triple positive breast cancer samples. Triple negative tissues displayed a signature characterized by *Aerococcus, Arcobacter, Geobacillus, Orientia,* and *Rothia*. Triple negative tissues displayed a signature characterized by with *Bordetella, Campylobacter, Chlamydia, Chlamydophila, Legionella,* and *Pasteurella*.  There were similarities in microbial signatures for the ER positive and Her2 positive breast cancer samples such as *Acinetobacter, Alcaligenes Anaplasma, Eikenella, Fusobacterium, Kingella, Lactococcus, Salmonella, Borrelia,* and *Klebsiell*a. | [(15)](https://www.zotero.org/google-docs/?PjRD0C) |
| To identify local breast tissue microbiome taxonomic differences between two racial groups | Fresh frozen breast tissue samples from Non-Hispanic Black women and non-hispanic White women  Normal (n=8)  Normal adjacent pair (n=11)  Tumor (n=64) | Human tissue microbiomes and isolated and analyzed via 16s rRNA gene sequencing.  Significance testing was done by analysis of variance using distance matrices (ADONIS) with treatment group at 999 permutations, linear discriminant LeFSe.  Alpha diversity was estimated with Shannon index, Chao1, Fisher’s alpha, and Richness metrics.  Phyla in breast tumors by stage was visualized with Spearman heatmaps. | All samples analyzed had the highest abundance of Proteobacteria and lower abundances of Firmicutes, Bacteroidetes, and Actinobacteria.  Breast tissues of non-Hispanic Black women showed higher abundances of genus *Ralstonia* than non-Hispanic White women.  Breast tissue microbiota differed with breast cancer stage. Genus *Bosea* increases with stage.  Triple negative breast cancer tissue was enriched in family *Streptococcaceae.* | [(17)](https://www.zotero.org/google-docs/?VlzxpL) |
| To determine which hypervariable region of the 16S rRNA gene includes the most useful information for breast tissue microbiota.  &  To find a less invasive form of tissue collection method (Core needle biopsies vs. surgical excision biopsies) for analysis and cancer diagnostic use. | Breast tissues from Mediterranean women (n=30)  Core needle biopsies (n=12)  Surgical excision biopsies (n=7) | Human breast tissue microbiomes were isolated and analyzed via 16s rRNA gene sequencing.  Alpha diversity between tumor and healthy samples with the two tissue collection methods was compared via Shannon index and observed OTUs  Relative abundance at the family and genus level is visualized via barplots | The V3 region of the 16S rRNA was the most informative for breast tissue microbiota.  No significant difference in reads and between OTUs between core needle biopsies and surgical excision biopsies.  There are more similarities between tumors and adjacent normal tissues.  Breast cancer samples had a lower relative abundance of *Methylobacterium* and higher relative abundance of *Ralstonia* compared to healthy tissues. | [(18)](https://www.zotero.org/google-docs/?xk98dh) |
| To investigate the anti-breast cancer potential of live, heat-killed cells (HKC), and the cytoplasmic fractions (CF) of *Enterococcus faecalis* and *Staphylococcus hominis* | MCF-7 breast cancer cell line.  MCF-10A non-malignant breast epithelial cell line  *Enterococcus faecalis* and *Staphylococcus hominis*  isolated from breast milk of Malaysian healthy women | MCF-7 cell line was treated with 25, 50, 100 and 200 μg/mL of live, HKC and CF of *Enterococcus faecalis* and *Staphylococcus hominis.*  Cytotoxicity was evaluated using MTT cell proliferation assay for 24, 48 and 72 hour time points.  The morphology of the treated cells was observed using fluorescence microscopy.  Cell cycle arrest and apoptosis were quantified and detected by flow cytometry. | Live, HKC, and CF of bacteria caused significant decrease in MCF-7 (up to 33.29%) cell proliferation.  Apoptosis via cell shrinkage and blebbing is observed in 34.60% of treated MCF-7 cells.  Significant anti-proliferative activity induced in MCF-7 through sub-G1 accumulation and decreased cell abundance in G0/G1 phase. | [(23)](https://www.zotero.org/google-docs/?0oIqLe) |
| To determine if *Fusobacterium nucleatum* affects breast cancer in a similar fashion to colorectal cancer | Formalin-fixed paraffin embedded breast cancer tissue samples (n=50) and frozen colon tumor (n=21)  Breast cancer tissue samples includes normal (n=35), benign (n=7), and malignant (n=64)  Mice used in study include vehicle group (n=8 per group) and *F. nucleatum*-infected group (n=10 per group) | Breast and colon tissue local microbiomes were isolated, 16S rDNA amplified, and sequenced with 16S rRNA. *Fusobacterium nucleatum* gDNA is then quantified.  1 × 10^6^ AT3 cells were injected into C57BL/6 mice and later injected with PBS vehicle or 5 × 10^7^ *F. nucleatum* ATCC 23726. Tumors were harvested to quantify abundance of NK cells via flow cytometry  1 × 10^5^ AT3 cells were injected into C57 SCID-beige mice and later injected with PBS vehicle or 5 × 10^7^ *F. nucleatum* ATCC 23726. The tumors were later harvested for weighing.  Breast cancer and normal breast tissues were compared by tissue microarray (HBre-Duc060CS-01) which is stained with FITC-labeled Gal-GalNAc-specific PNA lectin and Hoechst dye. | Human breast cancer progression is related to a Gal-GalNAc level increase.  *Fusobacterium nucleatum* gDNA levels are positively correlated to Gal-GalNAc level.  *F. nucleatum* in the breast suppressed the accumulation of tumor-infiltrating T cells and promotes tumor growth and metastatic progression. Promotion of tumor growth could be countered by antibiotic treatment. | [(25)](https://www.zotero.org/google-docs/?CGj7PW) |
| To characterize breast tissue microbiota and associate with tumor expression profiles | Breast tumor tissues (n=668), adjacent normal tissues (n=72) | Breast tissue RNA was sequenced via RNA sequencing. The microbiome was characterized by aligning non-human reads to bacterial genome/16S rRNA databases.  Differential abundance of microbial reads for most abundant operational taxonomic units was analyzed with limma. Host differential gene expression was analyzed with edgeR.  Correlation analysis was done by Spearman’s test. Gene set enrichment analysis was done by Fisher’s exact test. | Breast cancer tissues showed a higher abundance of *Proteobacteria.* Adjacent normal breast tissues showed a higher abundance of *Actinobacteria.*  Gene expression for epithelial to mesenchymal transitions was associated with *Listeria spp* abundance.  *H. influenza* correlated with proliferative pathway genes such as the G2M checkpoint, E2F transcription factors, and mitotic spindle assembly.  *S. pyogenes* correlated with *GUSBP4*, *GUSBP9*, and *GPA2* expression levels. | [(26)](https://www.zotero.org/google-docs/?JoYwqA) |
| To determine taxonomic breast tissue microbial signatures in intraoperatively obtained breast tissue samples | The breast tissue samples are collected from women who underwent surgery for breast cancer or benign diseases. (n=33) | Human breast tissue microbiomes are isolated and analyzed via PCR and 16S rDNA hypervariable tag sequencing and 16S rRNA sequencing.  Microbiome signature patterns are visualized with 2D ordination space plots.  PICRUSt was used to predict the functional metagenome.  Microbiome differences are identified through statistical analysis of alpha diversity (observed operational taxonomic units numbers and Shannon index) and beta diversity (UniFrac), and visualized with bar graphs and refraction curves. | There is a distinct breast tissue microbiome that differs from microbiota of external breast skin tissue, breast skin swabs, and buccal swabs.  There is a distinctly different microbial community profile between breast cancer tissues and benign breast disease tissues.The cancer tissues have a higher abundance of *Fusobacterium, Atopobium, Gluconacetobacter, Hydrogenophaga* and *Lactobacillus.*  Cancerous tissues showed reduced inositol phosphate metabolism, cysteine and methionine metabolism, glycosyltransferases, and fatty acid biosynthesis. | [(28)](https://www.zotero.org/google-docs/?bRlbGa) |
| To determine how a microbiome profile change can affect breast cancer development and aggressiveness | Needle biopsy breast tissue samples from Chinese breast cancer patients with malignant (n=72) and benign (n=22) breast diseases.  Grade I (n=7)  Grade II (n=36)  Grade III (n=13) | Human breast tissue microbiome are isolated and analyzed via 16S rRNA gene amplicon sequencing,  Alpha and beta diversity are visualized and compared with boxplots, Shannon index, and PCoA plots.  Taxonomic profiles are visualized with barplots. | There are distinct microbiome profiles in breast tissue of women between malignant cancer and benign breast diseases.  The cancer tissues are enriched in genus *Propionicimonas* and families Micrococcaceae, Caulobacteraceae, Rhodobacteraceae, Nocardioidaceae, Methylobacteriaceae. This may be specific to the ethnicity of this research group.  For samples that are more malignant (3 grades based on Nottingham Histologic Score system), relative abundances of family Bacteroidaceae decreased and genus *Agrococcus* increased.  Grade III tissue had an increase in glycerophospholipid and ribosome biogenesis, and a decrease in flavonoid biosynthesis. | [(32)](https://www.zotero.org/google-docs/?LaVZdh) |
| To determine the effects of *Lactobacilli* treatment on the  expression of HIF pathway genes in MDA-MB-231 cell line | MDA-MB-231 triple negative breast cancer cell line (no expression on estrogen and progesterone receptors and HER-2/Neu) | MDA-MB-231 cells are treated with *Lactobacillus crispatus* and *Lactobacillus rhamnosus* culture supernatant  RNA was isolated and the expression of HIF pathway genes (*HIF-1α, SLC2A1, VHL, HSP90, XBP1,* and *SHARP1*) were measured before and after treatment are analyzed using qRT-PCR  Cytotoxicity was evaluated using the MTT assay. | *Lactobacillus crispatus* and *Lactobacillus rhamnosus* supernatant both had cytotoxic effects on MDA-MB-231 cells.  *Lactobacillus crispatus* supernatant showed a higher cytotoxicity effect on MDA-MB-231 cells. *L. crispatus* significantly down-regulated *HSP90* and tumor supporsor genes (*VHL*, and *SHARP1*) expression*.*  *Lactobacillus rhamnosus* supernatant had a lower cytotoxicity effect on MDA-MB-231 cells. *L. rhamnosus* significantly down-regulated *HIF-1α, HSP90,* and *SLC2A1* expression.  The expression level of tumor suppressor genes *VHL* and *SHARP1* were also decreased in LCS treated cells. | [(49)](https://www.zotero.org/google-docs/?Qj0kLN) |
| To determine the modulation effects of neoadjuvant chemotherapy on tumor microbiome  &  To determine breast cancer signaling effects of  microbes | Breast tumor tissue of female patients who underwent neoadjuvant chemotherapy (n = 15)  Breast tumor tissue of women who had no therapy prior to surgery (n = 18) | Tumor microbiota DNA is isolated and population was identified using 16S rRNA-sequencing, and was confirmed using strained breast tissue microarrays  Effect of bacterial metabolites on breast cancer proliferation and Dox therapy responsiveness was evaluated using bacteria conditioned media.  Presence of *Pseudomonas* in breast tumor tissue was confirmed using IHC staining.  *In vitro* cell index was measured by electrical impedance every 6 hours.  Immune markers were analyzed with western blotting. | Breast tumor *Pseudomonas spp.* was significantly increased post chemotherapy administration.  Increased tumor abundance of *Brevundimonas* and *Staphylococcus* in primary breast tumors patients with distant metastases.  Treatment of cancer cells with *Pseudomonas aeruginosa* conditioned media and Dox differentially affected proliferation in a dose-dependent manner. | [(97)](https://www.zotero.org/google-docs/?YzswLo) |

**Table S2: Key research studies focused on the gut microbiome in breast cancer**

| Research Objective | Model Population | Methods | Key Findings | Reference Number |
| --- | --- | --- | --- | --- |
| To identify links between gut/fecal microbiome composition and breast cancer | Early stage cancer patients prior to treatment or surgery (n=31) | Bacterial DNA from fecal samples was isolated and amplified using qPCR, and sequencing using 16S rRNA sequencing.  Difference in bacterial abundance was determined using Wilcoxon or Kruskal-Wallis test. | Firmicutes, *F. prausnitzii, Blautia* spp., and *E. lenta* bacteria had a significantly lower abundance in the obese group than the normal BMI group.  *C. coccoides, F. prausnitzii*, and *Blautia* spp. had a significantly higher abundance in groups of clinical stage II and III than in clinical stages 0 and I. | [(34)](https://www.zotero.org/google-docs/?TjCOFd) |
| To determine if gut microbiome affects breast cancer pathogenesis. | Patients from Tumor Hospital of Guangxi Medical University in Nanning, China:  Premenopausal breast cancer patients (n=18)  Premenopausal healthy controls (n=25)  Postmenopausal breast cancer patients (n=44)  Postmenopausal healthy controls (n=46) | Illumina sequencing of DNA from fecal samples.  Gut microbiome diversity was calculated using the Shannon index and Chao1 indexes.  Relative genus abundance determined by JSD distance and a PAM clustering algorithm. | Breast cancer patients showed higher microbiome diversity compared to controls.  Premenopausal breast cancer patients and premenopausal controls did not show significant differences in relative species abundance.  Postmenopausal patients and postmenopause controls were significantly different in 45 bacterial species. 38 species were enriched while 7 were reduced.  Expression of high sensitivity C reactive proteins were positivity associated with *Acinetobacter radioresistens* and *Enterococcus gallinarum*  Estradiol levels are positively associated with *Shewanella putrefaciens* and *Erwinia amylovora.*  CD3^+^CD8^+^ T cell count was negatively associated with *Actinomyces sp. HPA0247.*  Genes encoding LPS biosynthesis, iron complex transport system, PTS system, secretion system, and beta-oxidation were enriched in postmenopausal breast cancer patients. | [(37)](https://www.zotero.org/google-docs/?h0MjlH) |
| To determine fecal microbiome profile differences between early stage breast cancer patients and controls | Healthy controls (n=30)  Breast cancer patients prior to cancer therapy (n=25) | DNA from fecal samples was subjected to qPCR and sequenced using V3-V4 16S rRNA sequencing.  Significant microbiome relative abundances were determined using ANOVA and Benjamini Hochberg adjustment and visualized as bar graphs. | Microbiome diversity was significantly lower in BC patients compared to healthy control.  BC patients displayed an increased abundance in Firmicutes, *Clostridium* cluster XIVa, and *Clostridium* cluster IV taxa.  BC patients displayed a decreased relative abundance of Bacteroidetes, *Bifidobacterium* sp., *Odoribacter sp., Butyricimonas sp.*, and *Coprococcus sp.* | [(38)](https://www.zotero.org/google-docs/?oqqr9K) |
| To determine if gut microbiome profile is different between those with malignant breast tumors and benign breast tumors | Chinese malignant breast cancer patients (n=83)  Chinese benign breast tumor patients (n=19) | Bacterial DNA in fecal samples was sequenced via 16S rRNA Illumina sequencing  Microbiome alpha and beta diversity and relative abundance was analyzed.  Gut microbiome functionality was determined by PICRUSt.  Significant biomarkers are identified using linear discriminant analysis (LDA) effect size (LEfSe) in relative genus abundances. | No significant difference in alpha and beta diversity between benign and malignant breast tumor groups.  Microbiome composition was different between benign and malignant breast tumor groups.  *Citrobacter* genus was significantly higher in the malignant breast tumor group.  Metabolic pathways were significantly different in malignant breast tumor group compared to the benign. | [(39)](https://www.zotero.org/google-docs/?jPDovQ) |
| To determine differences in gut microbiome function and structure between breast cancer and benign breast lesions patients | Breast cancer patients (n=27)  Benign breast lesions patients (n=22)  Healthy controls (n=21) | Gut microbiome composition was determined using 16S rRNA sequencing  Alpha diversity was computed using Sobs and Chao1 indexes  Beta diversity was compared using weighted and unweighted Unifrac distances | Breast cancer patients had lower alpha diversities than benign breast lesions patients and controls.  Benign breast lesions patients had alpha diversity indices than controls.  Beta diversity was significantly different between all groups  There was a higher abundance of *Porphyromonas* and *Peptoniphilus* in breast cancer patients.  There was a higher abundance of *Escherichia* and *Lactobacillus* in benign breast lesion patients. | [(40)](https://www.zotero.org/google-docs/?EDxb9k) |
| To determine the mechanics in which the gut microbiome modulates breast cancer development and how neutrophils play a role in etiopathogenesis in bacteria infected models | 3 month old FVB-Tg(C3-1-TAg)cJeg/JegJ female mice  12 weeks of *Helicobacter hepaticus* infected mice (n=51) | 3 month old FVB-Tg(C3-1-TAg)cJeg/JegJ female mice infected with *Helicobacter hepaticus.*  Mice were depleted of neutrophils through intraperitoneal injections of anti-Ly-6G antibody.  Mice bowel colonization is confirmed using PCR of cecum and stool samples post necropsy.  Histopathology is evaluated through hematoxylin and eosin tissue staining.  Immunohistochemistry is evaluated using rabbit polyclonal antibodies against Myeloperoxidase and heat induced antigen retrieval.  Histomorphometry is done by counting and imaging. | Tumor development is completely halted through antibody depletion in the *Helicobacter hepaticus* model.  Host neutrophil associated immune responses in the gut microbiome impacts cancer progression significantly.  Gut microbiome contributes to inflammation in the absence of inflammatory diseases. | [(46)](https://www.zotero.org/google-docs/?Jfx08f) |
| To determine how breast cancer is influenced by diet and genetic predisposition to breast cancer via modulation of the microbiota | Model 1: 8 week old outbred CD-1 mice given a westernized diet (increased breast cancer risk)  Model 2:  8 week old FVB strain erbB2 (HER2) mutant mice (mimics human breast cancers), fed a non-Westernized diet. | Female HER2/neu mutant mice treated with *Lactobacillus reuteri* ATCC-PTA-6475  Mice were depleted of CD25+ cells by using anti-CD25 antibody  CD4+ lymphocytes are sorted by hi-speed flow cytometry  Formalin fixed tissue histopathology of CD25+ cells is evaluated by hematoxylin and eosin stain analysis by a pathologist, Ki-67 is evaluated by DAB chromogen, Hematoxylin counterstain, and Mast cells are evaluated by toluidine blue stain  Tissue hyperproliferation state is evaluated with ki-67 specific immunohistochemistry | Breast cancer carcinogenesis in both groups is inhibited by *Lactobacillus reuteri* ATCC-PTA-6475 exposure.  CD4+CD25+ lymphocytes act as a protective mechanism post *Lactobacillus reuteri* ATCC-PTA-6475 exposure.  Transplanted *Lactobacillus reuteri* from one subject to another showed anticancer protection in the recipients. | [(47)](https://www.zotero.org/google-docs/?ELx90B) |
| To determine how *L. casei* ssp. modulate immune responses and mechanisms | 6-8 weeks old female BALB/c mice (n=30) | Test and control mice groups both received oral administration of *L. casei.*  Tumor antigen preparation is done using the protein dialysis method.  Hypersensitivity response is evaluated using a footpad induration.  Cytokine determination in spleen cell culture via ELISA.  NK cell mediated cytotoxicity evaluated using the lactate dehydrogenase assay. | Treatment of *L. casei* orally in mice significantly increased the production of IL-12, IFN-γ, and NK cell cytotoxicity in spleen cells.  Tumor growth rate in the *L. casei* mice decreased  Survival is significantly prolonged in *L. casei* treated mice compared to the controls. | [(48)](https://www.zotero.org/google-docs/?omc0NS) |
| To determine the antitumor activity of probiotics and fermented foods | 6-8 weeks old female BALB/c mice  T group: Tumor control with injection of tumor cells, no milk treatment  T/P+ group: Tumor injection and milk treatment group with *L. heveticus* R389  T/P- group: Tumor injection and milk treatment group with *L. heveticus* L89  P+ group: Milk treatment group only with *L. heveticus* R389  P- group: Tumor injection and milk treatment group with L. heveticus L89  NP group: no treatment control group, no tumor injection or milk treatment | Mice were injected with breast tumor cells and after 4 days received a 7 day treatment of with fermented milk with *L. helveticus.*  Cytokine concentrations were quantified using the ELISA assay.  Concentration of cytokine producing cells was determined by histological analysis of mammary gland tissue sections. | T/P+ group had an increased IgA and CD4 positive cells in breast tissue.  P+ and P- groups had no changes in immune cell or cytokine positive cell numbers.  T/P+ had a more pronounced increase in IL-10 and a decrease in IL-6 decreases than other groups. | [(50)](https://www.zotero.org/google-docs/?YrgDeh) |
| To determine the effect of exercise on breast cancer when co-treated with berberine (BBR) | 4T1,  MCF7, and MCF-10A cell lines (in vitro)  BALB/c 4T1 tumor-bearing mice grouped into four graphs: controls, exercise low, moderate, and high., | *In vitro* effects of exercise and BBR on breast cancer is observed using the MTT method, hoechst staining and cell morphology.  Anticancer molecular mechanisms studied using western blotting, intestinal microbiota characterization and SCFA detection, and qPCR.  Histological analysis and immunohistochemistry is done using hematoxylin and eosin stained tumor sections. | Exercise and BBR co-treatment slowed the progression of breast cancer significantly in 4T1 tumor-bearing mice.  The infiltration of NK cells increased in the BBR and exercise group compared to the control group  Immune factors and cytokines expression was regulated in the BBR and exercise group compared to the control group.  Level of SCFAs was significantly increased. This promoted apoptosis of 4T1 cells and changes in inflammatory factors *in vitro*.  Reduced expression of bcl-2 and XIAP in tumor tissues.  Increased expression of Fas, Fadd, Bid, Cyto-C, and Caspase-3/8/9 in vitro. | [(54)](https://www.zotero.org/google-docs/?7S5ORF) |
| To determine the antimetastatic and antiangiogenic effects of kefir in breast cancer | 5 to 6 weeks old female BALB/c mice (3 groups, n=7 per group)  4T1 breast cancer cell line | Anti-migration and anti-invasion effects of kefir are assessed using 4T1 cancer cells treated with kefir water in vitro.  Mice were injected with 4T1 cancer cells and treated orally with kefir water for 28 days  Cell apoptosis is visualized using the TUNEL assay  Mitotic cells are counted using microscopy using hematoxylin and eosin stained tumor tissue  Nitric oxide from samples are measured using the Griess reagent kit  Lipid peroxidation is measured using the malondialdehyde assay by thiobarbituric acid reaction  Blood serum cytokine detection is observed using Duoset Elisa assay  Amount of metastatic 4T1 cells are quantified using a clonogenic assay  Gene expression of selected genes is determined using qPCR  Multiple cytokines in sample is measured using the proteome profiler angiogenesis antibody array | Cytotoxicity is observed in 4T1 cells post kefir treatment  Tumor size and weight are significantly reduced, helper T cells increased by 5 fold, and cytotoxic T cells increased by 7 fold post kefir water treatment  Proinflammatory and proangiogenic markers were significantly reduced post kefir water treatment. | [(55)](https://www.zotero.org/google-docs/?oxokHb) |
| To determine gut microbiome profiles and functional pathways in premenopausal breast cancer patients. | Breast cancer patients (n=200) and controls (n=67) from Kaohsiung Medical University Chung-Ho Memorial Hospital | Bacterial DNA from fecal samples was isolated and sequenced using 16S rRNA sequencing.  Alpha and beta diversity were measured.  Microbiome composition was visualized using heat maps and correlation matrixes.  Gut microbiome functional pathways were predicted by PICRUSt2. | Significant reduction in α-diversity of premenopausal breast cancer patients.  Significant difference in β-diversity between breast cancer patients and controls.  *Bacteroides fragilis* was found in young premenopausal breast cancer patients.  *Klebsiella pneumoniae* was found in older women of postmenopausal statuses.  There was a significant difference in functional pathways observed between breast cancer patients and controls. | [(80)](https://www.zotero.org/google-docs/?GY08am) |
| To determine gut microbiome differences between postmenopausal pretreatment breast cancer patients , and healthy controls | Non-Hispanic white postmenopausal pretreatment breast cancer case patients (n=48), and control patients (n=48) | Bacteria DNA in fecal samples was isolated and 16S rRNA is sequenced with Illumina sequencing.  Estrogen concentration was quantified in urine using stable isotope dilution liquid chromatography/tandem mass spectrometry (LC-MS/MS).  Alpha diversity was evaluated using Chao1, Phylogenetic diversity (PD), and Shannon index. Beta diversity was calculated using UniFrac distances. | Postmenopausal pretreatment breast cancer patients had significantly different compositions, measured by alpha/beta diversity.  Postmenopausal pretreatment breast cancer patients had two fold higher mean urinary estrogens than controls (not significant).  Total estrogens positively  correlated with alpha-diversity in control patients but not postmenopausal pretreatment breast cancer patients. | [(83)](https://www.zotero.org/google-docs/?YVN84g) |
| To determine the influence of antibiotics on the gut microbiome and tumor growth | 8-10 week old female C57BL/6 mice | Mice microbiota is depleted prior to tumor cell injection  Gut microbe abundance is determined using metagenomic sequencing.  Tumor stromal signature cells are identified using single-cell transcriptomics.  Mast cell abundance is determined using histology analysis.  Tumors are analyzed using H&E, IHC, toluidine blue, and picro-sirius red staining and PyMT-BO1, BRPKp110, and EO771 tumor growth assays. | Gut microbiome dysbiosis caused by antibiotics significantly increased tumor progression.  Antibiotics caused reduced abundance of *Lactobacillus reuteri, Lachnospiraceae bacterium, Faecalibaculum rodentium*.  Antibiotics caused an increase in  cells with a stromal signature in tumors.  Antibiotics caused an increase in abundance of mast cells in the tumor stromal regions.  Mast cell stabilizer treatment and cromolyn decreased tumor growth in antibiotic treated animals but has no effect on the control group. | [(93)](https://www.zotero.org/google-docs/?fybXQZ) |
| To determine the association between the gut microbiome and breast cancer | Early stage breast cancer patients (n=76) post chemotherapy over the span of 10 years  Healthy Italian controls (n=54)  Subjects grouped by favorable (T1, Gr 1, N-, AJCC stage 1) and unfavorable prognosis ((T2-3, Gr 3, N+, AJCC stage II/III) | Alpha diversity was computed using observed OTU and the Shannon index.  Beta diversity was analyzed via NMDS.  Bacterial species were analyzed via variable importance plots and LeFSe. | Certain gut microbiome taxa were more abundant in breast cancer patients compared to healthy controls (*Streptococcus* genera*, Lachnospiraceae* family*, Veillonella* genus*, Bacteroides spp, E. ramosum, Enterobacteriaceae* family*, and Clostridiaceae* family).  Healthy controls were associated with *Methanobrevibacter* *smithii* archae, Eubacteriaceae family, *A. muciniphila, Defulfovibrio piger,* *Coprococcus* genus and *Collinsella* genus, *B. vulgatus*, Ruminococcaceae family  *B. uniformis* and *C. bolteae* were associated with worse disease outcomes.  Alpha diversity was not significantly different between all groups.  Beta diversity significantly differed between the groups. | [(94)](https://www.zotero.org/google-docs/?GrWn1a) |
| To determine association between gut microbiome, chemotherapy toxicity, and treatment response | Estrogen receptor positive breast cancer patients  Fecal samples collected before cyclophosphamide (AC) and docetaxel (D) (n=44), during AC (n = 43), during D (n = 29), and after AC-D treatment (n = 37) | Chemotherapy toxicity was assessed using scoring indices including: diarrhea, peripheral sensory neuropathy, hand-foot syndrome, fatigue, nausea, oral mucositis, vomiting, alopecia and constipation.  Bacterial DNA in fecal samples was isolated and V4 region of 16S rRNA was amplified and sequenced.  Bacterial relative abundance, alpha (Shannon and observed), and beta diversity were measured. | Species richness decreased during treatment overall.  Significant changes in *Lactobacillus, Ruminococcaceae NK4A214 group, Marvinbryantia, Christensenellaceae R7 group,* and *Ruminococcaceae UCG-005*  Significantly lower species richness in patients who had diarrhea during D treatment.  Shifts in microbial richness and abundance of specific bacterial taxa was observed during AC-D treatment. | [(95)](https://www.zotero.org/google-docs/?D4Wq68) |
| To determine if orally administered cancer therapeutics (Vismodegib) that target the Hedgehog (Hh) signaling pathway affects breast cancer | 5 weeks old female BALB/c immunocompetent mice injected with 4T1 luciferase-expressing cells  4T1 mice are split into 2 groups DMSO and Smo-i-treatment group | Bacterial DNA is isolated from fecal and cecum samples and V4 region of 16S rRNA is sequenced.  Microbiome relative abundance and alpha and beta diversity were measured.  Immunohistology analysis was done by tissue hematoxylin and eosin staining, as well as CD68 or myeloperoxidase staining. | Vismodegib increases the proliferation of CD8+ T cells.  The abundance of ruminal *Clostridium* decreased in very early tumor bearing mice and were at a constantly decreased levels in early and late tumor bearing mice.  The abundance of Clostridiales vadinBB60, *Lactobacillus gasseri*, Lachnospiraceae, and Anaeroplasma decreased in very early tumor bearing mice.  The abundance of Muribaculaceae increased in very early tumor bearing mice and was consistently increased in early and late tumor bearing mice.  Small shifts in alpha diversity in 4T1 mice from Smo-1 treatment.  Significant differences in beta diversity between DMSO and Smo-i-treatment groups. | [(96)](https://www.zotero.org/google-docs/?g280lI) |
| To determine if doxorubicin (Dox) can shift the gut microbiome.  &  To determine if the gut microbiome composition effects chemotherapy response | Female BALB/c mice (n=115)  Fecal transplant mice (n=40)  LPS study (n=30) | Mice were injected with 4T1 cells and treatment of Dox and/or antibiotics and a transplant of high-fat diet-derived fecal microbiota (HFD-FMT) or exogenous LPS.  Mice fecal DNA samples are isolated and metagenomic sequencing was performed.  Tracking, monitoring, and quantification of signals in tumors was done using in vivo bioluminescent imaging.  Immunohistochemistry was done using quantification on hematoxylin and eosin stained tissue.  LPS binding protein was quantified using ELISA. | The Dox responders, and antibiotics + Dox groups showed a reduction in tumor weight and metastatic burden.  Dox was associated with increased relative abundance of *Akkermansia muciniphila.*  HFD-FMT heightened tumor growth and decreased Dox responsiveness.  Dox nonresponders and FMT + Dox mice showed an increase of LPS in the blood plasma.  Intestinal inflammation and lung metastasis increased while Dox responsiveness decreased post exogenous LPS treatment. | [(99)](https://www.zotero.org/google-docs/?PsGaGU) |
